# Comparative Analysis of the Gut Bacterial Community in Laboratory-Reared and Seasonally Field-Released Larvae of the *Antheraea pernyi*

**DOI:** 10.3390/insects17010079

**Published:** 2026-01-09

**Authors:** Peng Hou, Li Liu, Ding Yang, Chuntian Zhang, Jianfeng Wang

**Affiliations:** 1 College of Life Science and Engineering, Shenyang University, Shenyang 110044, China; hp_0829@syu.edu.cn (P.H.); liujiali0229@163.com (L.L.); 2 College of Plant Protection, China Agricultural University, Beijing 110193, China; dyangcau@126.com; 3 College of Life Science, Shenyang Normal University, Shenyang 110034, China

**Keywords:** gut bacterial community, *Antheraea pernyi*, laboratory-reared, field-released, rearing environment and season

## Abstract

Analyzing the composition and structure of the gut bacterial community in *Antheraea pernyi* is essential for improving its economic traits, as well as for understanding gut bacteria–host interactions in lepidopteran insects. This study utilized the Illumina MiSeq PE 300 platform to conduct 16S rRNA gene sequencing for a comparative analysis of gut bacterial community in laboratory-reared and field-released (spring and autumn) *Antheraea pernyi* larvae of the same strain. The study revealed the specific effects of rearing environment and seasonal variation on the structural and functional dynamics of the larval gut bacterial communities. The composition of the dominant gut bacteria varied significantly with rearing environment and season. Laboratory-reared and spring field-released groups exhibited similar bacterial community structures, whereas the autumn field-released group showed a significant trend toward specialization, characterized by enrichment of specific bacterial taxa. Linear discriminant analysis effect size identified statistically significant biomarkers across samples. Taxonomic analysis revealed that Actinomycetota, Actinobacteria, Mycobacteriales, Dietziaceae, and *Dietzia* were characteristic of the gut bacteria profile in spring field-released, Lactobacillales, Enterococcaceae, and *Enterococcus* were enriched in the autumn field-released group, and the laboratory-reared group exhibited a relative dominance of Alphaproteobacteria. Functional prediction indicated that gut bacterial community structure likely influences its metabolic potential, which may suggest an adaptive response of the *Antheraea pernyi* to distinct ecological environments. This study provides important insights into the highly complex nature of insect-microbe interactions.

## Simple Summary

The insect gut bacterial community plays a crucial role in the dynamic physiological processes of the host. This study conducted a comparative analysis of gut bacterial communities among laboratory-reared, spring field-released, and autumn field-released larvae of *Antheraea pernyi* in the same strain. The study revealed that the structure and function of the gut bacterial community in *A. pernyi* are governed by both rearing environment and seasonal factors. The results contribute to enriching the understanding of gut bacterial community features under different rearing conditions and seasonal variations, providing insights and references for developing microbial resources in lepidopteran insects.

## 1. Introduction

The *Antheraea pernyi* (Chinese oak silkworm), belonging to the family Saturniidae within the order Lepidoptera, is an economically important insect that primarily feeds on *Quercus mongolica* leaves. China is the earliest country to have utilized and domesticated the oak silkworm, which, together with the *Bombyx mori*, constitutes one of the two pillars of Chinese silk civilization [1,2]. *A. pernyi* has extensive applications in diverse fields, including food, textiles, healthcare, biopesticides, and new materials. Its eggs can be utilized for rearing *Trichogramma* wasps to control the Asian corn borer (*Ostrinia furnacalis*) [3,4], whereas its cocoons can be reeled into silk textiles. Additionally, the protein-rich pupae and moths provide a valuable nutritional resource [5]. Currently, the *A. pernyi* industry has established a complete industrial chain encompassing egg production, rearing, cocoon procurement, silk reeling, weaving, deep processing, and sales [6,7].

Liaoning Province in China is a bivoltine region for *A. pernyi*, where the silkworms undergo two generations per year, classified as spring and autumn oak silkworms. The primary objective of rearing spring *A. pernyi* is to produce high-quality cocoons for the subsequent autumn generation. According to life cycle assessment data, *A. pernyi* farming utilizes 75–85% less land and reduces greenhouse gas emissions by 40–60% compared to traditional livestock production [8]. These characteristics enable the *A. pernyi* industry to be distinctive, sustainable, and of significant value-added potential. Furthermore, due to its unique advantages such as ease of rearing, short life cycle, and distinct phenotypic traits, *A. pernyi* serves as an important model insect for scientific research in Lepidoptera [9].

The insect gut bacterial community plays a crucial role in the dynamic physiological processes of the host and is considered an indispensable organ, significantly influencing host nutrition, immunity, and ecological adaptation [10,11,12]. These functions encompass the production of various digestive enzymes and the inhibition of pathogenic microbial invasions. Current studies indicate that the composition and structure of insect gut bacterial community is shaped by multiple factors, including host developmental stage, physicochemical conditions of the gut, dietary sources, habitat, and phylogenetic evolution, reflecting complex host–bacteria interactions [13,14]. The bacterial community in the gut of the *A. pernyi* significantly influences nutrient accumulation and growth during larval stages [15,16]. For example, *A. pernyi* larvae feed primarily on oak (*Q. mongolica*) leaves, which contain over 20% cellulose. While the larvae themselves do not produce endogenous cellulase, they rely on gut bacteria to degrade the cellulose, facilitating nutrient acquisition and development [17]. Therefore, analyzing the composition and structure of the gut bacterial community in *A. pernyi* is essential for understanding gut bacteria–host interactions in Lepidopteran insects and for improving *A. pernyi* economic traits. Existing studies have confirmed significant differences in gut microbial diversity across different larval instars of *A. pernyi* [15]. Moreover, host plants, rearing seasons, and genetic strains significantly affect the structure of the gut bacterial community [18]. However, the comparative analysis of the gut bacterial community between laboratory-reared and seasonally field-released *A. pernyi* larvae of the same strain has not yet been reported.

This study utilized the Illumina MiSeq PE 300 platform to conduct 16S rRNA gene sequencing for a comparative analysis of gut bacterial community diversity characteristics in laboratory-reared and (spring and autumn) field-released *A. pernyi* larvae. The study aims to enrich the understanding of gut bacterial community features under different rearing conditions and seasonal variations, and to provide insights and references for exploiting microbial resources in lepidopteran insects and utilizing beneficial bacteria in *A. pernyi*.

## 2. Materials and Methods

### 2.1. Experimental Insect Collection and Preparation

The experimental eggs of the *A. pernyi* were obtained from the bivoltine strain Ji Qing, developed by the Sericultural Science Research Institute of Jilin Province. Larvae reared under both laboratory and field conditions (spring and autumn) hatched from these eggs and underwent four molts to reach the fifth instar.

The laboratory-reared group was formed on 27 April 2025, at the Liaoning Provincial Key Laboratory of Urban Pest Management and Ecological Security. Larvae were fed daily with sufficient fresh leaves of 2–3-year-old Mongolian oak (*Q. mongolica*), which were surface-cleaned prior to feeding. Rearing conditions were maintained at 20–22 °C, 75–85% relative humidity, and a photoperiod of 14L:10D. The developmental period from egg hatching to the first day of the fifth instar lasted 48 ± 2 d.

The experiments on the spring and autumn field-released groups were conducted in a Mongolian oak plantation located in Qing Liangshan Town, Xiuyan County, Anshan City, a major sericultural region of Liaoning Province (N40°35′26.75″; E123°40′31.80″; altitude 298 m). This site has been planted with numerous 2–3-year-old Mongolian oaks, which provide a favorable ecological niche for research.

The spring field-released group was started on 29 April 2025, with eggs developed to the first-day fifth-instar larvae, lasting 45 ± 2 d. Environmental conditions were maintained at an average temperature of 22–25 °C, relative humidity of 80–85%, and a photoperiod of 14L:10D. The autumn field-released group was started on July 24, 2025, with eggs developed to the first-day fifth-instar larvae over 40 ± 2 d, under key parameters including an average temperature of 24–28 °C, relative humidity of 80–85%, and a photoperiod of 13L:11D.

The fifth instar is the final larval stage of *A. pernyi*. When provided with adequate nutrition and in a healthy state, larvae typically initiate cocooning approximately 14 days after entering this stage. For these experimental insects, all selected larvae were at 10 days into the fifth instar. Samples were designated as AP, WAPS, and WAPA: AP refers to laboratory-reared fifth-instar *A. pernyi* larvae, WAPS to spring field-released fifth-instar *A. pernyi* larvae, and WAPA to the autumn field-released fifth-instar *A. pernyi* larvae.

Insect samples were randomly selected and starved at room temperature for 48 h to empty their gut contents. This procedure established a standardized physiological baseline across all groups and minimized potential interference from food residues during subsequent microbial analysis. They were rinsed with a 4% NaCl solution pre-cooled to 4 °C until no visible foreign matter remained. Subsequent surface sterilization was performed using 75% ethanol for 60 s, repeated three times, followed by three washes with sterile water. The cleaned larvae were aseptically dissected in a laminar flow cabinet to obtain gut samples. The gut tissues were rinsed three times with 0.9% NaCl solution until no visible intestinal contents, transferred to 1.5 mL centrifuge tubes, flash-frozen in liquid nitrogen, and stored at −80 °C for future use.

For each group (AP, WAPS, and WAPA), gut samples were dissected from 30 larvae and pooled into three biological replicates (n = 10 guts per replicate), yielding a total of nine samples designated as AP1-AP3, WAPS1-WAPS3, and WAPA1-WAPA3.

### 2.2. Total DNA Extraction

Total bacterial community DNA was extracted from the larval gut according to the manufacturer’s instructions using the E.Z.N.A.^®^ Soil DNA Kit (Omega Bio-tek, Norcross, GA, USA). The DNA purity was assessed by measuring the OD260/OD280 ratio with a NanoDrop 2000 spectrophotometer (Thermo Scientific, Wilmington, NC, USA), and its integrity was confirmed by 1% agarose gel electrophoresis.

### 2.3. PCR Amplification and Sequencing

Using the total DNA of the sample as the template, PCR amplification of the target gene was performed with the universal bacterial 16S rRNA gene primers targeting the V3–V4 hypervariable region: forward primer 338F (5′-ACTCCTACGGGAGGCAGCAG-3′) and reverse primer 806R (5′-GGACTACHVGGGTWTCTAAT-3′). The PCR reaction was carried out in a total volume of 20 μL, containing 10 ng/μL template DNA, 4 μL of 5× FastPfu Buffer, 2 μL of 2.5 mM dNTPs, 0.8 μL each of the forward and reverse primers (5 μM), and 0.4 μL of FastPfu Polymerase. The PCR conditions were as follows: initial denaturation at 95 °C for 3 min; 29 cycles of denaturation at 95 °C for 30 s, annealing at 55 °C for 30 s, and extension at 72 °C for 45 s; followed by a final extension at 72 °C for 10 min, and a final hold at 4 °C (PCR instrument: ABI GeneAmp^®^ 9700, Union City, CA, USA). The PCR products were separated by 2% agarose gel electrophoresis, and target bands were excised and purified using the AxyPrep DNA Gel Extraction Kit (Axygen, Union City, CA, USA). The purified products were quantified using a Qubit^®^ 4.0 Fluorometer (Thermo Fisher Scientific, Waltham, MA, USA). Library preparation was performed using the NEXTFLEX Rapid DNA-Seq Kit, and sequencing was carried out on the Illumina MiSeq PE 300 platform by Majorbio BioTech Co., Ltd. (Shanghai, China).

### 2.4. Sequencing Data Processing

Raw paired-end reads were processed using fastp (version 0.23.4; https://github.com/OpenGene/fastp, accessed on 29 September 2025) for quality control, and assembled using FLASH (version 1.2.11; https://ccb.jhu.edu/software/FLASH/index.shtml, accessed on 30 September 2025). Detailed parameters and procedures followed the quality control and assembly workflow described by Hou et al. [15]. Subsequently, based on default parameters, the obtained high-quality sequences were denoised using the DADA2 plugin on the QIIME2 platform (version 2024; https://qiime2.org, accessed on 1 October 2025) to generate amplicon sequence variants (ASVs). To minimize the influence of sequencing depth on further diversity analysis, all samples were rarefied to 35,000 sequences according to the minimum data volume among samples. After rarefaction, the average sequence coverage of each sample still reached 99.09%. Taxonomic classification of ASVs was performed using the Naive Bayes classifier implemented in QIIME2 with a confidence threshold ≥ 0.7, based on the SILVA 16S rRNA gene database (version 138; https://www.arb-silva.de/, accessed on 3 October 2025), and sequences annotated as chloroplast or mitochondrial in all samples were removed. PICRUSt2 (version 2.2.0) (https://github.com/picrust/picrust2/, accessed on 3 October 2025) was used to predict the gene functions of bacterial communities from 16S rRNA sequencing data.

All 16S rRNA gene sequencing raw data were deposited in the Sequence Read Archive (SRA) under the accession numbers SRR36632118, SRR36632117, and SRR36632116 for AP; SRR36632115, SRR36632114, and SRR36632113 for WAPS; and SRR36632112, SRR36632111, and SRR36632110 for WAPA.

### 2.5. Bioinformatic Analysis

All bioinformatics analyses of the sequencing data were performed on the Majorbio Cloud Platform (https://cloud.majorbio.com; accessed on 4 October 2025). Species rarefaction curves were constructed based on ASV data, with curves from different samples distinguished by color. Alpha diversity indices were calculated using MOTHUR (version 1.30.2; http://www.mothur.org/wiki/Calculators; accessed on 4 October 2025), including the Chao index, Ace index, Shannon index, and Simpson index. Intergroup comparisons were performed using the Kruskal–Wallis test. The Bray–Curtis dissimilarity was used to measure differences in bacterial community structure among samples. Based on this, principal coordinate analysis (PCoA) was performed to visualize the overall separation among sample groups. Additionally, analysis of similarity (ANOSIM) was applied to test the significance of differences in community structures across different groups. Linear discriminant analysis effect size (LEfSe) (http://huttenhower.sph.harvard.edu/galaxy/; accessed on 5 October 2025) was employed to identify taxa that exhibited significant differences and were most likely to explain the disparities in bacterial communities (LDA score > 4, *p* < 0.05). Subsequently, metabolic functional prediction of the bacterial communities was performed based on KEGG gene functional annotation.

## 3. Results

### 3.1. General Sequencing Data Results

**Figure 1:**
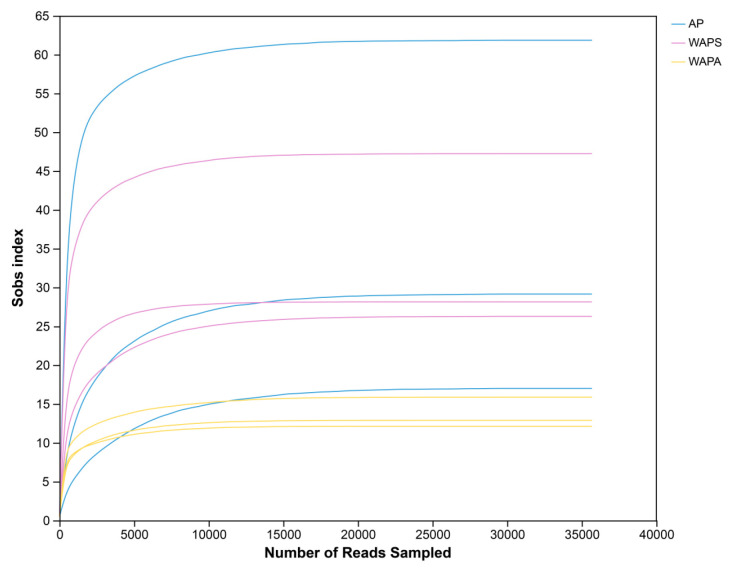
Rarefaction curve of the gut bacterial community in AP/WAPS/WAPA.

The V3–V4 region of the 16S rRNA genes from the samples (AP, WAPS, and WAPA) was sequenced on the Illumina MiSeq PE 300 platform. After quality control, filtering, and denoising, a total of 532,890 valid reads were obtained, with an average length of 413 bp. Using DADA2, valid reads were compared at single-nucleotide differences, clustered, and then sequences aligning to chloroplast and mitochondrial references were removed. A total of 155 ASVs were obtained, ranging from a minimum of 11 in sample WAPA3 to a maximum of 62 in sample AP2 (Table 1). Among these, the number of ASVs in AP is similar to that in WAPS, whereas WAPA shows significantly fewer ASVs compared to the other two groups. The rarefaction curves (Figure 1) revealed that as sequencing depth increased, the number of observed species (at the ASV level) in each sample group gradually stabilized and eventually reached a plateau. This indicates that the current sequencing depth is sufficient to uncover most bacterial diversity in the samples. A total of 12 phyla, 16 classes, 43 orders, 65 families, and 85 genera were annotated and statistically identified from the valid reads.

### 3.2. The Composition and Diversity of the Gut Bacterial Community in AP/WAPS/WAPA

**Figure 2:**
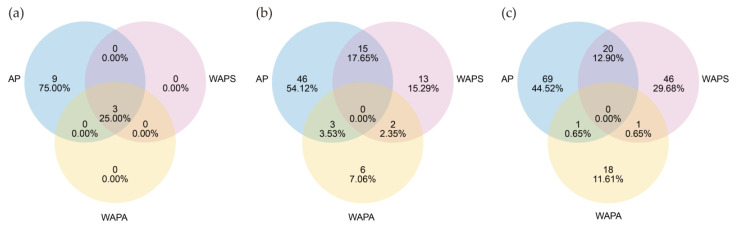
Venn analysis of gut bacterial communities in *A. pernyi* larvae across samples: (**a**) at the phylum level; (**b**) at the genus level; (**c**) at the ASV level.

Venn analysis showed that all three groups shared only three phyla. The laboratory-reared larvae of *A. pernyi* (AP) harbored 9 unique bacterial phyla, unlike field-released larvae (WAPS, WAPA), which had none (Figure 2a). At the genus level, the AP group contained the highest number of bacterial genera (64), among which 46 were unique to AP, 15 were shared with WAPS, and 3 were shared with WAPA. WAPS and WAPA shared 2 bacterial genera (Figure 2b). At the ASV taxonomic level, the numbers of ASVs unique to AP and WAPS were 69 and 46, accounting for 44.52% and 29.68% of the total bacterial ASVs, respectively. The number of ASVs shared between these two groups was 20, representing 12.90% of the total bacterial ASVs, which was higher than that observed between other groups (Figure 2c). Overall, no bacterial taxa were common to AP, WAPS, and WAPA at either the genus or ASV level, while a greater number of shared bacterial taxa were observed between AP and WAPS.

**Figure 3:**
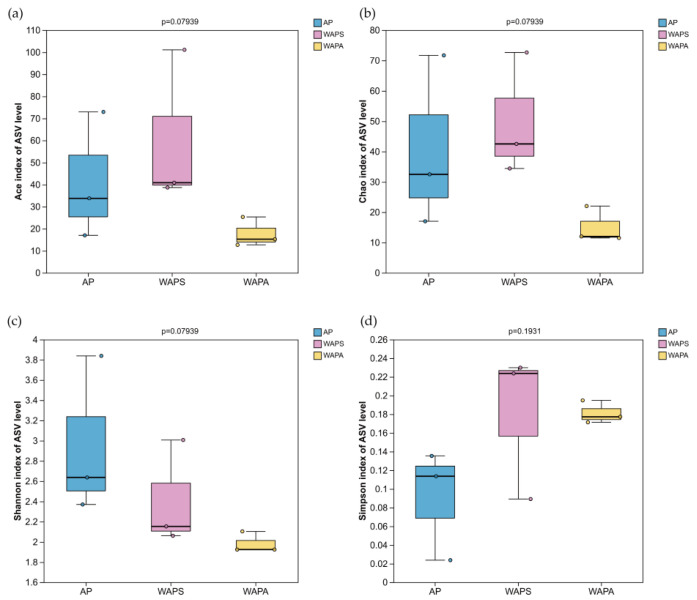
Kruskal–Wallis test of the gut bacterial community’s diversity in *A. pernyi* larvae across samples: (**a**,**b**) boxplot of species richness (number of ASVs) assessed by the ACE and Chao1 indices; (**c**,**d**) boxplot of bacterial community diversity assessed by the Shannon and Simpson indices.

Analysis of Alpha diversity indices (Table 2) showed that WAPS had the highest ACE and Chao indices, while WAPA had the lowest, indicating that WAPS possessed the highest species richness in the gut bacterial community, followed by the AP group, with WAPA having the lowest. Regarding the bacterial community diversity, the AP had the highest Shannon index and the lowest Simpson index, suggesting the greatest diversity. In contrast, WAPA had the lowest Shannon index and the highest Simpson index, indicating the lowest diversity. Notably, although differences in the alpha diversity were observed among the three groups (AP, WAPS, and WAPA), the Kruskal–Wallis test indicated no statistically significant differences in bacterial community richness or diversity (Figure 3).

**Figure 4:**
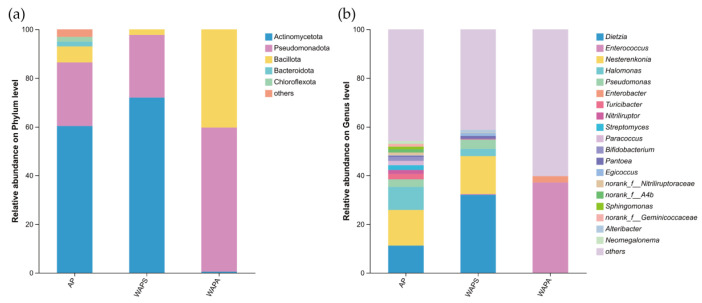
Composition of gut bacterial community in samples at different taxonomic levels: (**a**) at the phylum level; (**b**) at the genus level.

Taxonomic analysis revealed that the gut bacterial community of *A. pernyi* larvae was dominated at the phylum level by Actinobacteria (44.28%), Pseudomonadota (37.01%), and Bacillota (16.36%). However, the composition of the dominant gut bacteria varied significantly with rearing environment and season. In the AP group, the dominant phyla (relative abundance > 1%) consisted of Actinobacteria (60.32%), Pseudomonadota (26.17%), Bacillota (6.50%), Bacteroidota (1.95%), and Chloroflexota (1.95%). Both the WAPS and WAPA groups were dominated by Actinobacteria, Pseudomonadota, and Bacillota. Among these, Actinobacteria were most abundant in WAPS (72.03%), whereas Pseudomonadota (59.17%) and Bacillota (40.32%) were more prevalent in WAPA (Figure 4a). At the genus level, the composition of the gut bacterial community in *A. pernyi* larvae also differed across rearing environment and season. The AP group exhibited 13 bacterial genera exceeding 1% relative abundance, including *Dietzia*, *Nesterenkonia*, *Halomonas*, *Pseudomonas*, *Turicobacter*, *Nitriliruptor*, *Streptomyces*, *Paracoccus*, *Bifidobacterium*, *Pantoea*, etc. Notably, *Nesterenkonia* (14.63%) and *Dietzia* (11.22%) showed relatively high abundances in this group. In the WAPS group, *Dietzia* (32.03%) was the predominant genus, followed by *Nesterenkonia* (15.61%). In contrast, the WAPA group was overwhelmingly dominated by *Enterococcus* (37.07%), with *Enterobacter* (2.60%) as the second most abundant genus (Figure 4b).

**Figure 5:**
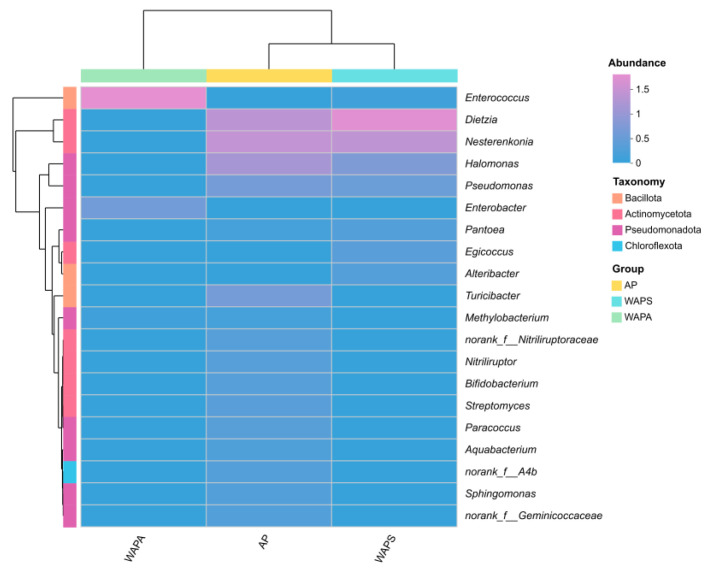
Clustered heatmap of the top 20 abundant gut bacterial communities in samples. Note: Rows and columns represent the samples and the dominant genera, and the right side of the figure is the value represented by the color gradient.

Further clustered heatmap analysis of the 20 most abundant bacterial genera revealed that the AP group and the WAPS group clustered on the same branch, whereas the WAPA group formed a distinct separate branch. This clustering pattern indicated that the dominant gut microbiota in the AP group and the WAPS group exhibited highly similar taxonomically specific variations, while the bacterial structure of the WAPA group showed significant differences (Figure 5).

**Figure 6:**
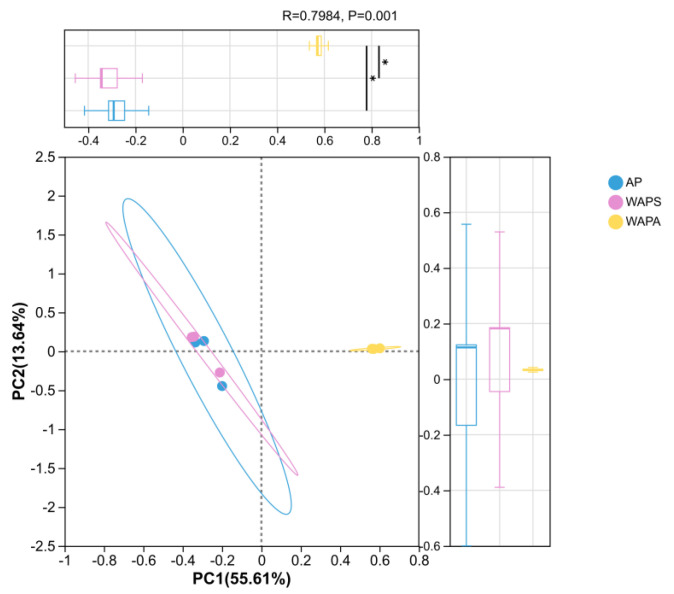
PCoA plot showing the variation in gut bacterial communities of *A. pernyi* larvae across samples. Principal components (PCs) 1 and 2 accounted for 55.61% and 13.64% of the variance. Note: * indicates significant differences (*p* < 0.05).

Principal coordinates analysis (PCoA) based on Bray–Curtis dissimilarity, combined with ANOSIM statistical testing, revealed significant differences in the gut bacterial community structure among the three sample groups (R = 0.7984, *p* = 0.001) (Figure 6). Samples in the WAPA group exhibited a high degree of spatial clustering and uniform distribution, indicating minimal intra-group variation in bacterial structure and good reproducibility. Moreover, this group was distinctly separated from the other two groups, further supporting the uniqueness of its gut bacteria composition. In contrast, partial overlap was observed between the AP and WAPS groups, while both were clearly separated from the WAPA group, suggesting that the bacterial community structures of these two groups are relatively similar.

### 3.3. The Differences and Specificity of Gut Bacterial Community in AP/WAPS/WAPA

**Figure 7:**
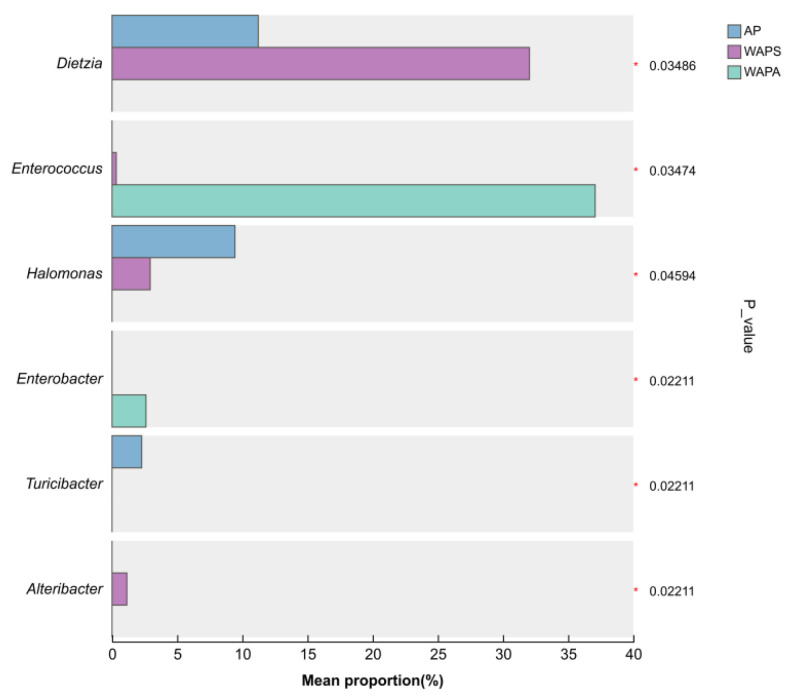
Significance analysis of the top 30 bacterial genera across *A. pernyi* larvae samples. Note: * indicates significant differences (*p* < 0.05).

Significance testing for samples revealed that six of the top 30 most relatively abundant bacterial genera exhibited significant alterations (Figure 7). *Dietzia* and *Halomonas* represented higher proportions in the AP and WAPS, showing statistically significant differences compared to the WAPA group (*p* < 0.05). *Enterococcus* and *Enterobacter* were most abundant in the WAPA and were significantly elevated relative to the other two groups (*p* < 0.05). *Turicibacter* was significantly enriched in the AP (*p* < 0.05), whereas *Alteribacter* demonstrated a higher abundance in the WAPS.

**Figure 8:**
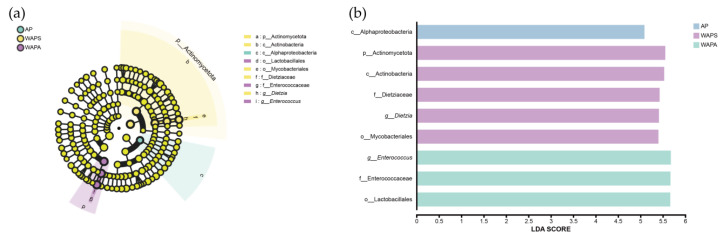
LEfSe analysis of the gut bacterial community of *A. pernyi* larvae across samples: (**a**) cladogram of bacterial taxa enriched across samples; (**b**) LDA discriminate histogram. Note: Phyla (p), Class (c), Order (o), Family (f), Genus (g).

Using linear discriminant analysis effect size (LEfSe) to identify specific bacterial biomarkers across samples (LDA > 4), we found that Actinomycetota were significantly enriched in the WAPS group at the phylum level. At the class level, Actinobacteria were significantly enriched in WAPS, while Alphaproteobacteria were predominantly enriched in the AP group. At the order level, Mycobacteriales showed significant enrichment in WAPS, whereas Lactobacillales were mainly enriched in WAPA. At the family level, Dietziaceae contributed substantially to the bacterial composition of WAPS, and Enterococcaceae primarily influenced the bacterial community of WAPA. At the genus level, *Dietzia* was identified as a key biomarker in WAPS, and *Enterococcus* played a dominant role in WAPA (Figure 8a). Collectively, *Actinomycetota*, *Actinobacteria*, *Mycobacteriales*, *Dietziaceae*, and *Dietzia* were characteristic of the gut bacteria profile in WAPS; Lactobacillales, Enterococcaceae, and *Enterococcus* were enriched in WAPA; and the AP group exhibited a relative dominance of Alphaproteobacteria (Figure 8b).

### 3.4. The Functional Prediction of the Gut Bacterial Community in AP/WAPS/WAPA

**Figure 9:**
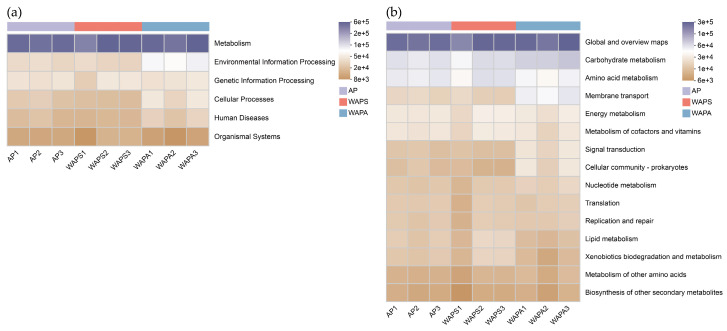
KEGG pathway annotations of gut bacterial community functions in *A. pernyi* larvae across samples: (**a**) KEGG primary pathway annotation level; (**b**) KEGG secondary pathway annotation level. Note: The color gradient of the blocks was utilized to illustrate changes in functional abundance across different groups. A higher value corresponds to greater functional abundance.

Functional prediction analysis based on the KEGG database using PICRUSt2 revealed that metabolism was the dominant function at the level of primary metabolic pathways across all sample groups, followed by genetic information processing and environmental information processing (Figure 9a). Further analysis of secondary metabolic pathways indicated that the global and overview maps were the most abundant in the AP, WAPS, and WAPA groups. Metabolic functions, including carbohydrate metabolism, amino acid metabolism, energy metabolism, and metabolism of cofactors and vitamins, were also highly abundant across all samples. Additionally, the WAPA group exhibited higher potential functional abundance in membrane transport, signal transduction, cellular community—prokaryotes, and nucleotide metabolism. In contrast, the WAPS group showed relatively higher levels of a potential function in lipid metabolism and xenobiotic biodegradation and metabolism (Figure 9b).

## 4. Discussion

Insects primarily acquire gut bacterial communities through feeding or contact with their surrounding environment. These bacterial communities play a crucial role in their physiological processes and adaptation to environmental stresses [19,20,21]. This study characterized the composition and differences of the gut bacterial community in the same strain of *A. pernyi* larvae reared under different feeding conditions and seasons. The study showed that rearing environment and seasonal variation have specific effects on the gut bacterial community of *A. pernyi* larvae.

This study revealed that the gut bacterial communities of *A. pernyi* larvae remain relatively stable at the phylum level across different rearing environments and seasons, primarily comprising Actinomycetota, Pseudomonadota, and Bacillota. This structural consistency aligns with previous reports in other lepidopteran insects, although variations were observed in the relative abundances of specific bacterial groups [20,22]. Among microorganisms, Actinomycetota are the most significant contributors to natural products [23]. Actinomycetes are known to produce approximately 70% of all microbial bioactive compounds discovered to date, including antibiotics, immunosuppressants, and enzyme inhibitors [24]. The dominance of Actinobacteria in the insect gut can be attributed to long-term coevolution with the host, and their contribution to host food digestion, nutrient acquisition, and pathogen defense [25,26]. Nevertheless, research on insect-associated actinomycetes remains at an initial stage. The isolation of actinomycetes from insects represents a key strategy for the discovery of novel species and bioactive metabolites. In this study, we showed that the Actinomycetota are predominant in the AP and WAPS groups, thereby establishing a valuable model for the isolation of insect-associated actinomycetes and the exploration of their secondary metabolites. Pseudomonadota and Bacillota were the predominant phyla in the WAPA group. Pseudomonadota are widely distributed in diverse environments, including animals, humans, and air, and frequently occur as dominant gut bacteria in many insects, such as *Pieris rapae* [27], *Choristoneura fumiferana* [28], and *Plutella xylostella* [29]. These bacteria aid in the decomposition of plant secondary metabolites, including terpenoids, alkaloids, glycosides, and phenolic compounds, thereby enhancing the host’s utilization of food resources and supporting normal growth and development [30]. Bacillota have been extensively implicated in key host physiological processes, including carbohydrate metabolism, amino acid metabolism, and membrane transport pathways [31].

At the genus level, the bacterial community structures of AP and WAPS exhibited similarity, with a substantial overlap in shared bacterial genera and ASVs. Clustering heatmap of abundant gut bacterial communities and PCoA analysis further supported the structural resemblance between the two groups. This finding may be attributed to similarities in food sources and environmental conditions. Specifically, there is convergence in environmental conditions such as developmental duration, photoperiod, and temperature/humidity between the AP and WAPS groups. Additionally, both groups forage on emerging tender leaves of Mongolian oak, which exhibit similar cellulose content, phenolic compound accumulation, and nutritional composition. The gut bacterial communities of both AP and WAPS were predominantly composed of genera such as *Dietzia* and *Nesterenkonia*. *Dietzia* can degrade complex organic compounds, including long-chain alkanes, aromatic compounds, sterols, and terpenoids. Its abundance in the AP and WAPS guts may facilitate the breakdown of toxic or indigestible phytochemicals into harmless or utilizable nutrients for the host. *Nesterenkonia* exhibits tolerance to high salinity and alkaline conditions, which may be associated with the alkaline environment in the midgut of *A. pernyi* larvae. Whether it participates in the physiological activities of the host remains to be further investigated.

In contrast, the gut bacterial community structure of WAPA underwent significant changes. Clustered heatmap analysis of abundant bacterial genera indicated that the WAPA group formed a distinct, separate branch. In PCoA, the WAPA group was also distinctly separated from the other two groups.

The WAPA exhibited significant differences in gut bacterial community composition and structure, with the dominant genera being *Enterococcus* and *Enterobacter*. This community profile closely resembled the microbial detection results from the intestinal tract of *B. mori* larvae. It was also consistent with previous reports indicating abundant *Enterococcus* in the midgut of late-instar *Spodoptera frugiperda* larvae [32].

In autumn, leaves undergo aging, leading to increased cellulose and secondary metabolite content, while changes in photoperiod and temperature differences may reduce the adaptability of certain microbial taxa, thereby altering the structure of the gut microbiota in *A. pernyi* larvae. *Enterococcus*, a facultative anaerobic bacterium, can gain a competitive advantage due to its strong stress tolerance, effectively suppressing the colonization and growth of exogenous pathogens through nutrient and spatial competition, thereby maintaining gut microbial homeostasis [33,34]. Previous studies have confirmed that *Enterococcus* significantly inhibits the germination of *Nosema bombycis* in vitro [35]. *Enterococcus* in the *Lymantria dispar* acidifies the local environment to facilitate its own colonization, helping the host suppress pathogenic toxins that are activated under alkaline conditions [36,37]. Previous studies have successfully isolated an alkali-resistant, heat-tolerant, safe, and non-toxic strain of lactic acid bacteria from *Enterococcus* in the gut of *B. mori.* This strain can co-aggregate with pathogenic bacteria and shows potential for development as a microbial agent or intestinal probiotic [38]. Therefore, based on the structural specificity of the WAPA bacterial community, further exploration and development of *Enterococcus* strains from the gut of *A. pernyi* larvae are warranted. Significantly, WAPA displayed a significantly larger body size and higher food intake than WAPS. *Enterobacter* can assist the host in digesting certain food components and perform detoxification functions [39]. The *Enterobacter* in WAPA likely contributes to the degradation of phenolic compounds that accumulate due to seasonal variation, thereby facilitating detoxification, promoting digestion and nutrient absorption, and ultimately supporting enhanced host growth and development.

Furthermore, LEfSe identified specific bacterial biomarkers across different groups, with WAPS exhibiting specific bacterial taxa, including five microbial taxonomic units belonging to the Actinomycetota. In contrast, WAPA was primarily characterized by Lactobacillales and Enterococcaceae, while Alphaproteobacteria dominated in AP. These differences likely reflect adaptive responses of the gut bacterial communities to host metabolic demands under varying rearing environments and seasonal conditions. Previous studies have indicated that the relatively weak constitution of *B*. *mori* may be associated with fluctuations in the abundance of Enterococcus and Pseudomonas in their gut, with certain key bacteria also showing significant correlations with traits such as pupal weight and cocoon shell weight [40]. Lactobacillus can activate the innate immune system of *B. mori*, enhancing larval survival rates [41,42]. As a typical probiotic, Lactobacillus exhibits strong carbohydrate fermentation capabilities, contributing to the maintenance of the intestinal barrier, immune regulation, and pathogen suppression [43]. In this study, Lactobacillales and Enterococcaceae were significantly enriched in WAPA. It can be inferred that compared to the spring field-released, the autumn field-released *A. pernyi* larvae are exposed to more complex environmental conditions, such as parasitic insect infestations and greater diurnal temperature variations. These factors may disrupt the balance of the larval gut microbiota, thereby enhancing their immunoregulatory capacity and adaptability.

Functional prediction across the samples indicated that the gut bacterial communities play an important role in maintaining the intestinal microenvironmental homeostasis and regulating host growth and development. These communities exhibited high activity in fundamental physiological processes such as carbohydrate metabolism, amino acid metabolism, energy metabolism, as well as metabolism of cofactors and vitamins. This may be attributed to the fact that the samples originated from the same strain with stable genetic traits and were fed on leaves of the same plant species with similar profiles. The WAPA group showed significant enrichment in functions such as membrane transport, signal transduction, cellular community-prokaryotes, and nucleotide metabolism. It is inferred that the gut microbiota of *A. pernyi* larvae enhanced their ability to sense external signals and transport substances to maintain metabolic homeostasis in response to complex external environments and seasonal shifts in dietary composition during autumn. In contrast, the WAPS group exhibited higher activity in lipid metabolism, xenobiotic biodegradation, and metabolic pathways, which may be related to their consumption of fresh spring leaves. These functional differences further illustrate how distinct growth environments and seasonal variations shape the metabolic potential of gut bacteria in *A. pernyi*, reflecting functional specialization in bacterial community adaptation to environmental conditions. Although PICRUSt2 has been widely used for predicting functional genes of gut microbiota, predictions based on the 16S rRNA gene V3–V4 region remain indirect and cannot directly confirm the actual functions of these microbial communities or their direct effects on host growth and development. Therefore, further validation through metagenomics or other experimental methods is still required.

## 5. Conclusions

Understanding the characteristics of the gut bacterial community of the *A. pernyi* is crucial for comprehensively elucidating its ecology and improving its economic traits. This study conducted a comparative analysis of gut bacterial communities among laboratory-reared, spring field-released, and autumn field-released larvae of the same strain of *A. pernyi.* The results revealed that the structure and function of the gut bacterial community in *A. pernyi* are governed by both rearing environment and seasonal factors. Laboratory-reared and spring field-released groups exhibited similar bacterial community structures, whereas the autumn field-released group showed a significant trend toward specialization, characterized by enrichment of specific bacterial taxa, likely due to changes in food quality and climatic conditions. This likely reflects the directional selection exerted by seasonal variations and food quality on dominant gut bacterial communities. Functional prediction indicated that gut bacteria play a key role in host environmental adaptation, and bacterial community structure likely influences its metabolic potential, which may suggest an adaptive response of the *A. pernyi* to distinct ecological environments. This study provides important insights into the highly complex nature of the lepidopteran insect gut bacterial community. Future research will integrate phenotypic data with measured functional assays to clarify the specific mechanisms by which key bacterial taxa affect *A. pernyi* growth, immunity, and stress resistance.

## Figures and Tables

**Table 1:** Basic information on the valid sequencing of the gut bacteria 16S rRNA.

Samples		Valid Reads	Average Length (bp)	Max Length (bp)	Min Length (bp)	Number of ASV
AP	1	62,320	406	430	262	17
	2	56,434	407	430	225	62
	3	65,707	406	527	267	29
	Total	184,461	406	527	225	108
WAPS	1	44,184	423	473	210	47
	2	51,755	409	528	201	26
	3	68,551	408	431	246	28
	Total	164,490	413	528	201	101
WAPA	1	54,974	419	540	208	12
	2	55,958	419	516	203	17
	3	73,007	418	446	202	11
	Total	183,939	419	540	202	40

Note: AP (1, 2, 3), three replicates of laboratory-reared fifth-instar *A. pernyi* larvae; WAPS (1, 2, 3), three replicates of spring field-released fifth-instar *A. pernyi* larvae; WAPA (1, 2, 3), three replicates of the autumn field-released fifth-instar *A. pernyi* larvae.

**Table 2:** Alpha diversity indices of the gut bacterial community in AP/WAPS/WAPA.

Samples	Richness		Diversity	
	ACE	Chao	Shannon	Simpson
AP	41.25 ± 28.72	40.40 ± 28.20	2.95 ± 0.78	0.09 ± 0.06
WAPS	60.22 ± 35.43	47.87 ± 26.15	2.41 ± 0.52	0.15 ± 0.04
WAPA	17.75 ± 6.68	15.17 ± 5.92	1.98 ± 0.10	0.21 ± 0.01

Note: Data are mean ± SE. AP, laboratory-reared fifth-instar *A. pernyi* larvae; WAPS, spring field-released fifth-instar *A. pernyi* larvae; WAPA, autumn field-released fifth-instar *A. pernyi* larvae.

## Data Availability

All metadata presented in this study are deposited in BioProject: PRJNA1395343. For further inquiries, please contact the first author.
